# INTRODUCTION TO THE STUDY OF MUSCLE, MOBILITY, AND AGING (SOMMA) AND PRELIMINARY RESULTS

**DOI:** 10.1093/geroni/igac059.830

**Published:** 2022-12-20

**Authors:** Steven Cummings, Anne Newman

## Abstract

SOMMA is a new cohort study conducted at Wake Forest and University of Pittsburgh, coordinated by the San Francisco Coordinating Center, with management of biological specimens by Adventist Health Research Institute. We aim to discover the biological basis of mobility disability and have collected tissues and measurements for future studies of the biology of human aging. SOMMA has enrolled ~875 older men and women (aged □70 years), and will follow-up participants by phone and in-person every six months for up to three years. An additional cohort of younger adults (aged 30-69) is being enrolled. In this symposium, we will describe the underlying conceptual construct for the study; the baseline characteristics of the cohort; recruitment techniques to enroll participants. We will present preliminary data from ~425 participants, including the association between mitochondrial energetics and muscle function (strength and power); the interrelationship between physical and cognitive measures; and the association of D3Cr muscle mass with strength and walking speed. Finally, we will describe how to obtain data and specimens for analyses and ancillary studies.

